# Aggressive Head and Neck Squamous Cell Carcinoma in the Setting of *GATA2* Deficiency

**DOI:** 10.1002/hed.28117

**Published:** 2025-02-21

**Authors:** Brejjette Aljabi, James A. Stewart, Kirk Withrow, Carissa M. Thomas

**Affiliations:** ^1^ Heersink School of Medicine, University of Alabama Birmingham Alabama USA; ^2^ Department of Otolaryngology‐Head and Neck Surgery University of Alabama at Birmingham Birmingham Alabama USA

**Keywords:** *GATA2* deficiency, head and neck cancer, human papillomavirus, squamous cell carcinoma

## Abstract

**Background:**

*GATA2* deficiency is a rare genetic disorder associated with hematologic, infectious, and neoplastic complications. We report a case of a patient with *GATA2* deficiency who developed aggressive squamous cell carcinoma (SCC) of the head and neck, an atypical manifestation of this condition.

**Methods:**

A 34‐year‐old Hispanic male, a nonsmoker, presented with a large, exophytic right facial mass. Biopsy revealed HPV‐negative SCC. Computed tomography (CT) showed a right periorbital mass invading the nasal cavity and a contralateral mass in the left parotid extending into the masticator space. The patient underwent extensive surgery, including right orbital exenteration, total rhinectomy, partial glossectomy, left radical parotidectomy, excision of the left mandibular condyle, and bilateral neck dissections. Reconstruction included a left temporalis muscle flap, internal fixation of the left zygoma, intermaxillary fixation, and a staged anterolateral thigh (ALT) free flap for the right facial defect. He developed postoperative *Pseudomonas* surgical site infections in the left face and ALT donor site and was treated with antibiotics and antifungals based on intraoperative culture results. The patient's postoperative course involved multiple interventions to address complications and support recovery. He developed bilateral local and regional recurrences rapidly after surgery and ultimately elected for palliative care.

**Results:**

Due to the aggressive nature of this case in a young, nonsmoking patient, combined with the atypical infections, genetic testing was performed for immunodeficiency syndromes. He was ultimately diagnosed with *GATA2* deficiency.

**Conclusion:**

This case highlights the aggressive nature of SCC in the context of *GATA2* deficiency and underscores the importance of genetic testing in patients with unusual malignancy presentations and suspected immunodeficiency. Genetic testing in the patient's children allows for early diagnosis of *GATA2* deficiency and provides an opportunity for curative intervention through hematopoietic stem cell transplantation.

## 1 Introduction

*GATA2* deficiency is a rare genetic disorder stemming from haploinsufficiency of the *GATA2* gene and represents a syndrome with heterogeneous clinical presentations [1]. *GATA2* is critical for regulating hematopoietic stem cells and promotes cellular differentiation in various tissues [2]. Heterozygous *GATA2* mutations manifest as hematologic, infectious, pulmonary, dermatologic, neoplastic, and vascular/lymphatic diseases, but immunodeficiency remains the hallmark feature of *GATA2* deficiency. Patients exhibit drastic immune cell cytopenias, which increase susceptibility to viral infections and solid tumors [3]. *GATA2* deficiency can present at any age and with varying severity, making it challenging to achieve timely diagnosis and appropriate management. The optimal therapeutic approach is hematopoietic stem cell transplantation (HSCT), but this treatment remains an area of evolving investigation [4]. By exploring this clinical case, we aim to enhance our understanding of *GATA2* deficiency and contribute to improved patient care and outcomes in this complex disorder.

We report a case of a 34‐year‐old Hispanic male who presented due to intolerable pain associated with a rapidly growing facial mass. The patient underwent surgical excision with a staged microvascular free flap reconstruction, but his postoperative course was complicated by recurrent *Pseudomonas* infections, slow wound healing, and rapid recurrence despite clear margins. Genetic testing revealed *GATA2* deficiency. This case report is noteworthy for presenting an unusual occurrence of *GATA2* deficiency associated with aggressive squamous cell carcinoma (SCC) in the head and neck—an atypical manifestation given the predominantly hematologic nature of the cancers related to *GATA2* deficiency.

### 1.1 Case 1

**FIGURE 1:**
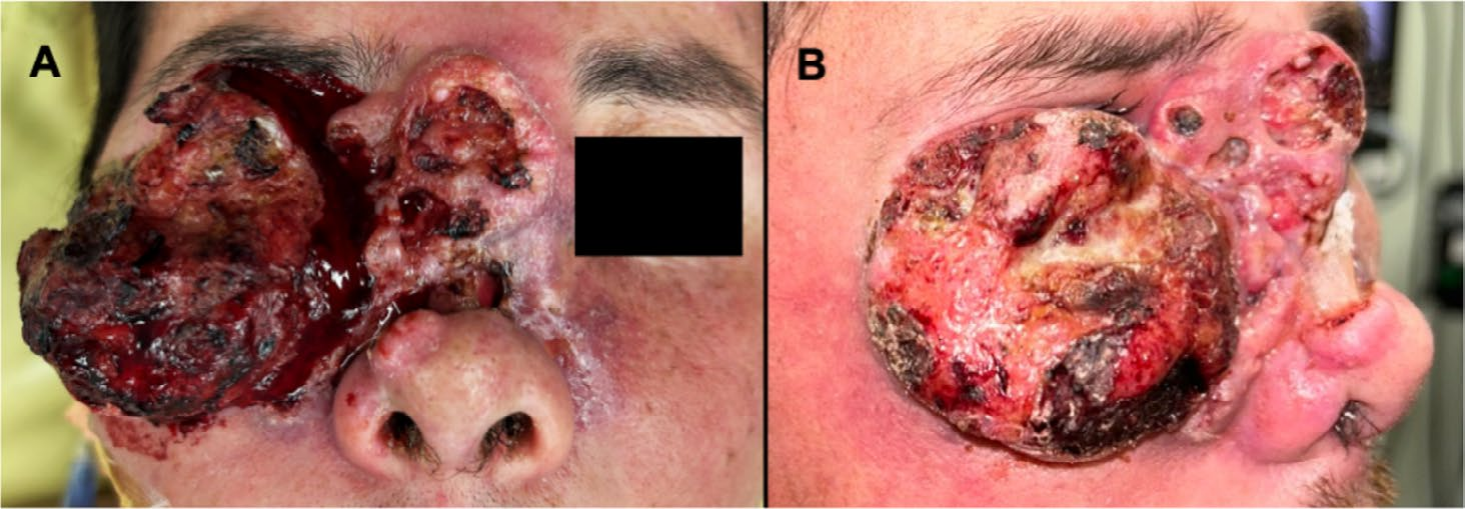
Anterior (A) and lateral (B) views of the exophytic, human papillomavirus‐negative squamous cell carcinoma involving the right cheek, nasal dorsum, right lower eyelid, obstructing the right eye, and extending onto the left medial cheek and encroaching upon the left medial canthus. This mass also infiltrated the nasal cavity.

This is a 34‐year‐old Hispanic male, a nonsmoker, with a past medical history significant for verrucous carcinoma of the fifth digit, extensive human papillomavirus (HPV)‐driven verruca vulgaris on all extremities, leukopenia, and anemia of unknown etiology, and an unknown vasculitis of the stomach. He presented to the head and neck clinic for evaluation of a large, exophytic right facial mass. Figure 1. Before presentation, he had a biopsy at an outside facility demonstrating HPV‐negative SCC. His physical exam was significant for an advanced, exophytic mass involving the right cheek, nasal dorsum, and right lower eyelid that obstructed the right eye, extended onto the left medial cheek, and encroached upon the left medial canthus. This mass also infiltrated the nasal cavity. Extraocular muscles were intact, and the patient denied vision changes or diplopia. A computed tomography (CT) with contrast demonstrated an extensive, heterogeneously enhancing right periorbital soft tissue mass with invasion to the nasal cavity. This resulted in chronic deformity of the nasal bone and suspected involvement of the right lacrimal gland. Bilateral orbits and intraconal spaces did not show tumor involvement. Additionally, a similar heterogeneously enhancing soft tissue mass was seen occupying the left parotid and infiltrating into the masticator space and left lateral pterygoid muscle. A fine needle aspiration biopsy of the left parotid and masticator space mass was non‐diagnostic but eventually confirmed SCC. A separate lesion on the right lateral tongue demonstrated high‐grade dysplasia.

**FIGURE 2:**
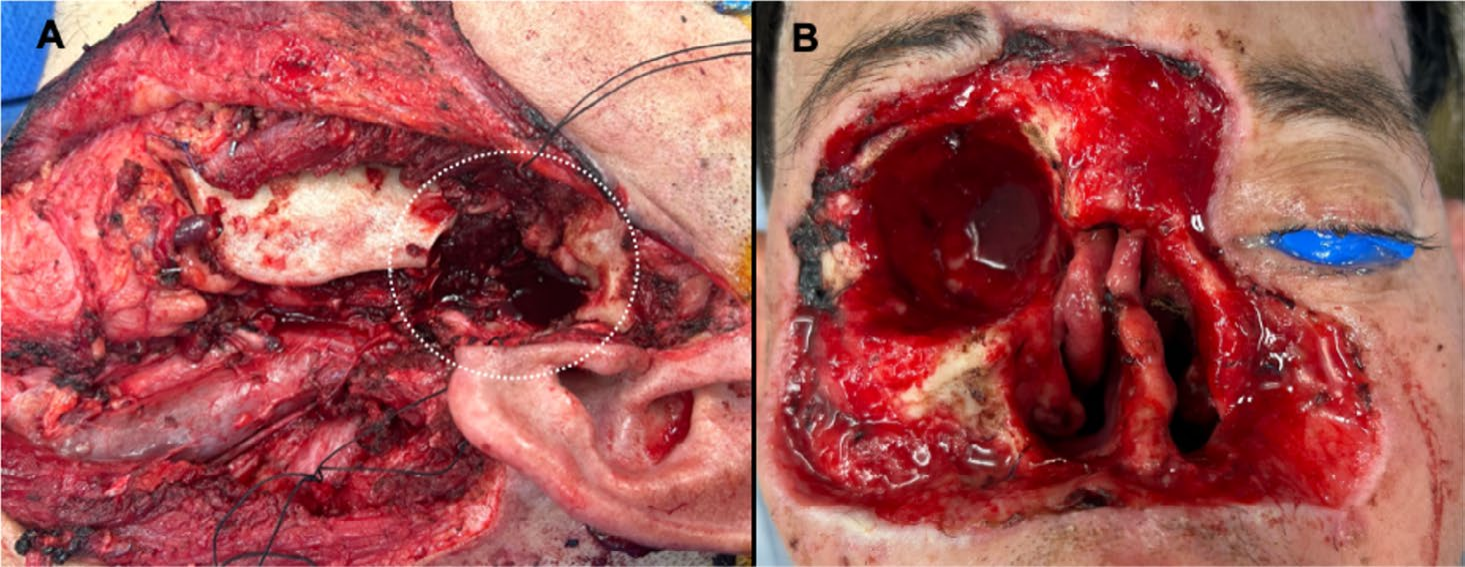
Post‐resection, there were defects involving (A) the left parotid, masseter, and temporomandibular joint (white dotted circle) as well as (B) the right orbit, nose, and wide local excision of the facial skin from the right cheek, glabella, and left medial cheek.

**FIGURE 3:**
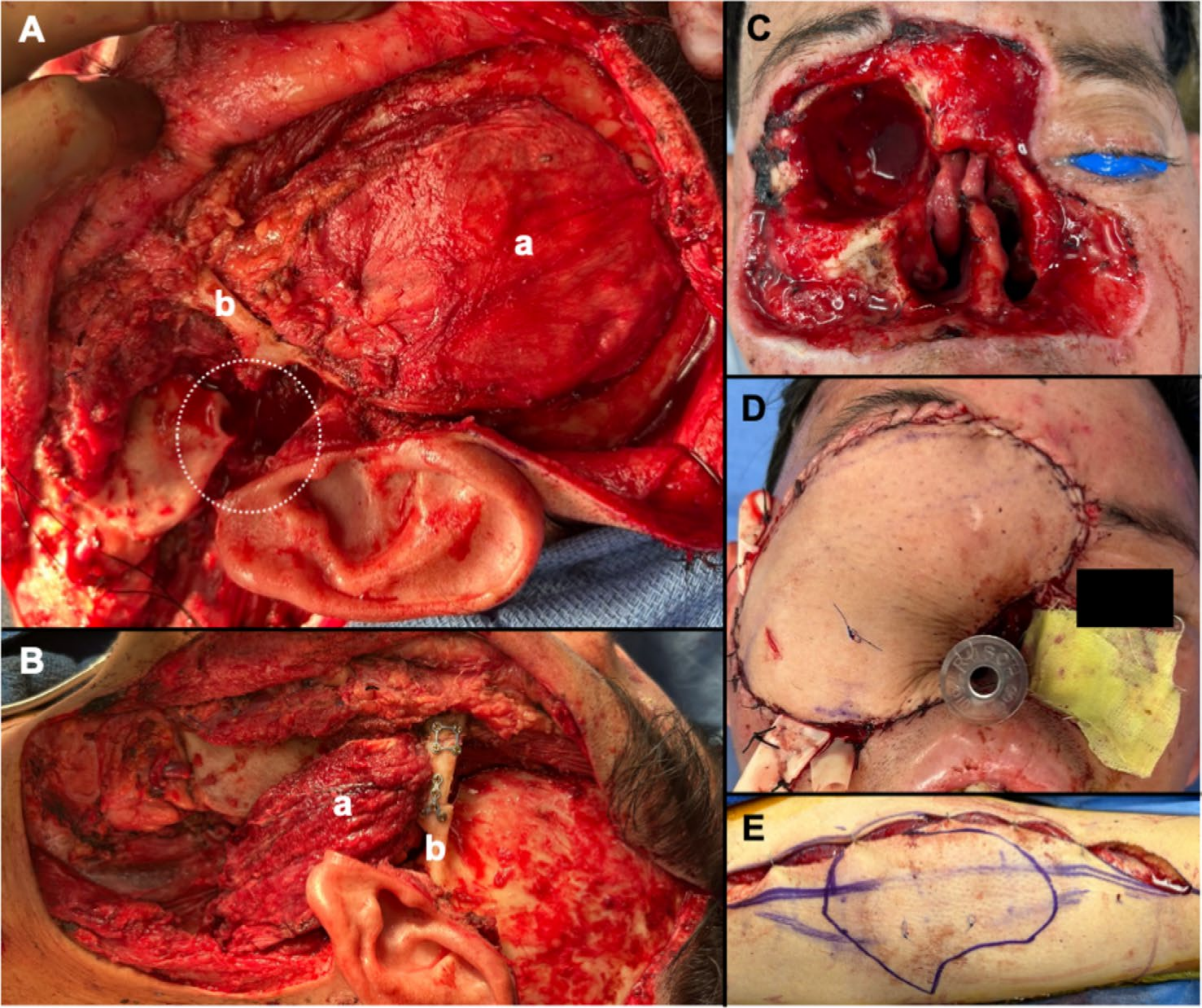
Reconstruction for the left‐sided defects (A, B) involved rotating the left temporalis muscle (a) underneath the zygoma (b) to fill the temporomandibular joint space. The patient was placed in intermaxillary fixation with bands to guide his occlusion. The zygoma (b) was cut and underwent open reduction and internal fixation to assist with the rotation of the temporalis flap. Staged reconstruction of the right‐sided defects (C–E) involved a templated anterolateral thigh free flap. The total rhinectomy defect would have been reconstructed with a prosthesis once treatment was completed.

The patient was presented at a multidisciplinary head and neck tumor board with a consensus recommendation for surgical resection, as neoadjuvant systemic therapy would not alter the extent of the surgical resection. His surgery included a wide local excision of the right face, right orbital exenteration, total rhinectomy, partial glossectomy, left radical parotidectomy, excision of the left mandibular condyle, and left neck dissection. Figure 2. Intraoperatively, the decision was made to stage reconstruction of the right face due to the extensive nature of the resection, the desire to confirm clear margins, and the need for reconstruction at the left radical parotidectomy and condylar defect. Reconstruction for the left‐sided defect involved a left temporalis muscle flap, open reduction and internal fixation of the left zygoma (divided to allow passage of the temporalis muscle into the inferior defect), and intermaxillary fixation with bands. Figure 3. The right facial defect was covered with a Xeroform bolster. In the immediate postoperative period, the patient became hypotensive, requiring pressor support and two units of packed red blood cells. He was monitored in the surgical intensive care unit overnight. Vasopressor support was weaned, the patient stabilized, and the remainder of the postoperative course was uncomplicated. The patient was discharged on postoperative day (POD) 4 with the plan for reconstruction of the right face in 10 days.

The patient presented 1 day after discharge to the emergency room with left facial edema, erythema, and tachycardia stemming from a postoperative wound infection. He was also hyponatremic and had elevated liver transaminases, which were attributed to dehydration after evaluation by the hospitalist team. Exploration of the left parotidectomy wound bed in the OR revealed extensive purulence, and appropriate drains were placed to allow for bedside wound irrigation. Antibiotic coverage was broadened from ampicillin/sulbactam to vancomycin and cefepime. Intraoperative culture data was positive for Methicillin‐resistant *Staphylococcus aureus* , *Pseudomonas aeruginosa* , and *Candida albicans* . Fluconazole was added, and additional washouts were performed.

Despite the recent left face and neck surgical site infection, the decision was made to proceed with reconstructive surgery for the right facial defect with the goal of not delaying adjuvant radiation and chemotherapy treatment. A right parotidectomy, right neck dissection, and right anterolateral thigh (ALT) free flap were performed. Figure 3. On POD4, a sudden increase in edema at the ALT donor site was noted, and a hematoma was evacuated, followed by placement of negative pressure wound therapy (NPWT). The ALT donor site subsequently developed a *P. aeruginosa* infection. During the 28‐day hospital course, serial neck and leg washouts were performed, and eventually, NPWT was applied to both the left facial and neck wound and the right leg wound. Multiple subspecialties were consulted due to the unusual nature of this case, including infectious disease, dermatology, and genetics. Based on their recommendations, the patient was tested for genetic immunodeficiency syndromes that increase susceptibility to HPV infections, such as WHIM syndrome, and an Invitae genetic panel was performed. He was diagnosed with *GATA2* deficiency, an autosomal dominant disorder, felt to be the underlying cause of his symptoms and disease course. Genetic testing was recommended for all his children since they could be treated with HSCT if they carried the disease‐causing variant.

**FIGURE 4:**
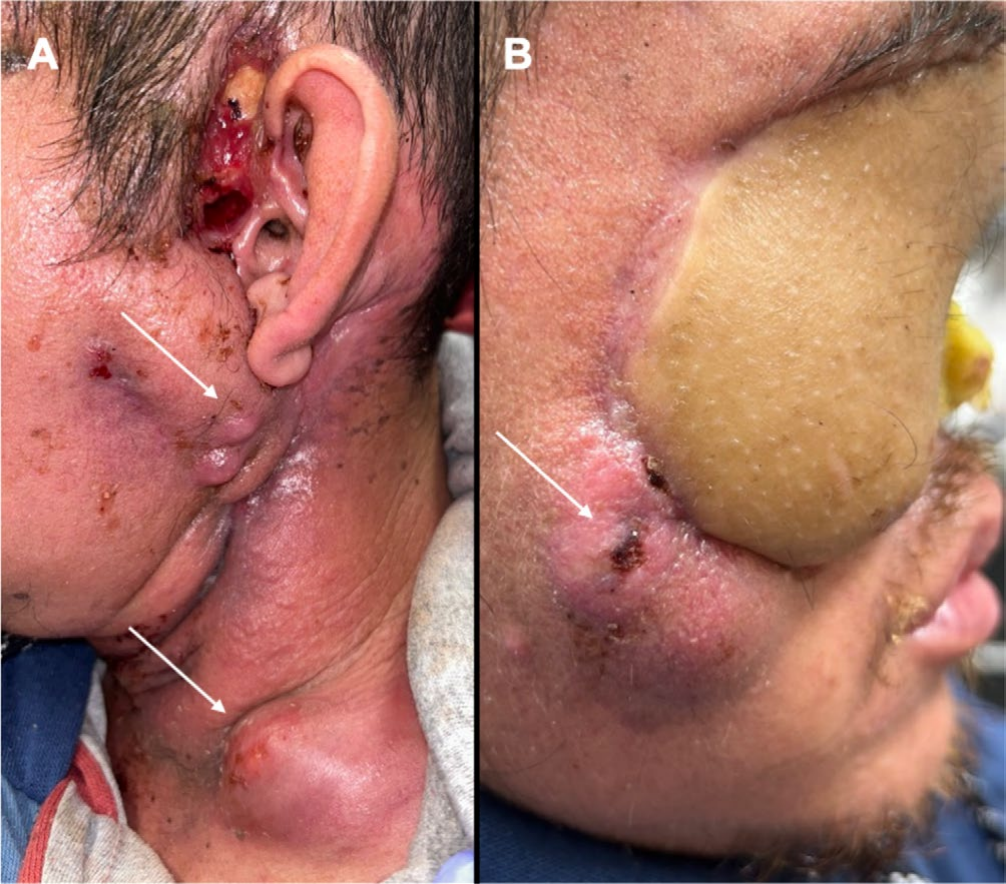
Rapid recurrence 46 days after the initial surgery despite clear margins (A, B). White arrows indicate areas of recurrent squamous cell carcinoma.

Approximately 10 days after discharge, the patient was noted to have a new right facial lesion along the lateral, inferior border of the ALT flap reconstruction involving the native cheek. Fine needle aspiration was positive for SCC, confirming recurrence 46 days after the initial surgery. One week later, he developed another recurrence in the left neck. Figure 4. He was evaluated for adjuvant therapy, and the decision was made to pursue immunotherapy combined with chemotherapy (carboplatin and paclitaxel) due to a combined positive score (CPS) of 15. Before the initiation of adjuvant treatment, the patient decided to pursue palliative care and declined all treatment options.

## 2 Discussion

A unique case of an aggressive head and neck squamous cell carcinoma that was ultimately discovered to be in the setting of *GATA2* deficiency is reported. *GATA2* encodes a zinc finger transcription factor and exhibits expression in various hematopoietic cells, particularly in early progenitors, megakaryocytes, and mast cells. *GATA2* plays a crucial role in the proliferation and maintenance of hematopoietic stem cells. Beyond hematopoietic tissues, *GATA2* expression extends to endothelial cells, the central nervous system, the placenta, the fetal liver, and the fetal heart [2]. *GATA2* has also been shown to modulate the behavior of angiogenic cells by directly binding to the VEGFR2 promoter to regulate its transcription, implicating *GATA2* in angiogenesis and wound healing [5]. Mutations in *GATA2* are inherited in an autosomal dominant fashion or acquired de novo in the germline, resulting in a loss of function in the mutated allele. The mutation is passed down in an autosomal dominant fashion, regardless of inheritance mode, highlighting the importance of familial genetic testing. The resulting haploinsufficiency gives rise to a clinical syndrome termed *GATA2* deficiency [6].

*GATA2* deficiency presents a broad array of clinical manifestations, such as myelodysplastic syndrome/acute myeloid leukemia (MDS/AML), pulmonary alveolar proteinosis (PAP), congenital lymphedema, sensorineural hearing loss, viral warts, and skin cancers, including basal cell carcinoma, squamous cell carcinoma, and malignant melanoma. Despite this diversity, the defining feature remains immunodeficiency. Profound cytopenias of monocytes, dendritic cells, B cells, and NK cells are highly specific for *GATA2* deficiency, although the reason for the selective deletion of these lineages remains unclear. Cytopenias heighten susceptibility to infections such as HPV (presenting as recalcitrant warts, condylomas, or dysplasia), *Mycobacterium avium* complex (MonoMAC), and Nontuberculous Mycobacteria (NTM) [5]. Notably, solid tumors associated with *GATA2* deficiency are linked to underlying viral infections, particularly HPV and EBV [7]. Interestingly, the patient presented had HPV‐driven verruca vulgaris but HPV‐negative SCC. One other case of HPV‐positive SCC of the oropharynx in the setting of *GATA2* deficiency was observed by Spinner et al. [5]. Finally, a recent report described Merkel cell carcinoma (MCC) in the setting of germline *GATA2*‐deficiency but did not specify if the Merkel cell polyomavirus virally drove the MCC [8]. NK cell deficiency, which plays a pivotal role in mediating antiviral and antitumor effects, likely contributes to viral infections and subsequent neoplastic transformations.

Workup for immune deficiency or genetic abnormality should be considered early in patients with recurrent, severe, or refractory infections, especially if the infections are chronic, caused by unusual or opportunistic organisms, or result in organ damage. Infections presenting in early childhood or in the context of a family history of persistent or unusual infections should also prompt further testing [9, 10]. For example, recurrent and recalcitrant warts should raise suspicion for an inborn error of immunity, with early genetic testing improving outcomes, particularly in conditions like *GATA2* deficiency [11]. Additionally, in cases of malignancy, a genetic evaluation may be warranted in patients with atypical or aggressive presentations without known risk factors and concomitant infections [12].

In addition to *GATA2* deficiency, other genetic conditions should be considered in the differential diagnosis of aggressive SCC and recurrent infections. These include *STAT1* gain‐of‐function mutations, which lead to chronic mucocutaneous candidiasis, viral infections, and an increased risk of SCC in the context of HPV; *DOCK8* deficiency, characterized by recurrent and severe infections, eczema, and an elevated risk of HPV‐associated SCC; WHIM syndrome, caused by mutations in the *CXCR4* gene, presenting with recurrent bacterial infections, severe HPV infections leading to warts and SCC, and neutropenia due to myelokathexis; *CTLA4* haploinsufficiency, presenting with recurrent infections, autoimmunity, and an increased malignancy risk (including SCC) due to impaired regulatory T cell function; and Fanconi anemia, which shares overlapping features with *GATA2* deficiency, including aggressive SCC and recurrent infections, but is more commonly associated with congenital disabilities, bone marrow failure, and early‐onset cancers, particularly head and neck SCC [13, 14]. Genetic testing and clinical evaluation are essential for accurate diagnosis and management, as treatment strategies, including HSCT, vary depending on the underlying genetic abnormality.

The onset of *GATA2* deficiency ranges from early childhood to late adulthood, presenting clinically with a spectrum from asymptomatic to life‐threatening infections, leukemia, and respiratory failure [15]. Early identification can guide patient counseling, such as in understanding the hereditary nature of the disease, the need for genetic testing in family members, and the potential for organ‐specific complications. A confirmed genetic diagnosis directly impacts clinical management, especially when deciding whether to initiate HSCT or pursue gene‐specific therapies [6]. Allogeneic HSCT is the only curative therapy for hematological complications of *GATA2* deficiency and has been shown to eradicate clonal malignancy, restore normal hematopoiesis, clear underlying infections, and improve pulmonary function. However, *GATA2* deficiency is a newly defined disease, and strategies and outcomes have yet to be fully elucidated [16, 17]. It is unknown whether HSCT is feasible or effective once an aggressive malignancy has developed. Identification of genetic disorders such as *GATA2* deficiency is still important in the setting of malignancy; however, as identification is essential for improved counseling, treatment decisions, and prevention in family members.

In shedding light on this case, we aim to underscore the critical importance of recognizing and understanding the diverse clinical manifestations of this condition, emphasizing the need for early diagnosis and tailored therapeutic approaches to improve patient outcomes. Highlighting this case is also important to emphasize the role of familial genetic testing. Early recognition would allow for curative intervention with an HSCT in descendants who inherited *GATA2* deficiency.

## Ethics Statement

The University of Alabama at Birmingham Institutional Review Board has reviewed and approved this case report under protocol number IRB‐300000348. As the patient is deceased, consent was not obtained. However, all identifying information has been removed to ensure confidentiality, and any images included have been edited to block or obscure identifying features.

## Conflicts of Interest

The authors declare no conflicts of interest.

## Data Availability

Data sharing is not applicable to this article as no new data were created or analyzed in this study.

## References

[1] A. P. Hsu , L. J. McReynolds , and S. M. Holland , “GATA2 Deficiency,” Current Opinion in Allergy and Clinical Immunology 15, no. 1 (2015): 104–109. 25397911 10.1097/ACI.0000000000000126 PMC4342850

[2] C. Vicente , A. Conchillo , M. A. García‐Sánchez , and M. D. Odero , “The Role of the GATA2 Transcription Factor in Normal and Malignant Hematopoiesis,” Critical Reviews in Oncology/Hematology 82, no. 1 (2012): 1–17. 21605981 10.1016/j.critrevonc.2011.04.007

[3] M. A. Spinner , L. A. Sanchez , A. P. Hsu , et al., “GATA2 Deficiency: A Protean Disorder of Hematopoiesis, Lymphatics, and Immunity,” Blood 123, no. 6 (2014): 809–821. 24227816 10.1182/blood-2013-07-515528 PMC3916876

[4] M. Parta , N. N. Shah , K. Baird , et al., “Allogeneic Hematopoietic Stem Cell Transplantation for GATA2 Deficiency Using a Busulfan‐Based Regimen,” Biology of Blood and Marrow Transplantation 24, no. 6 (2018): 1250–1259. 29412158 10.1016/j.bbmt.2018.01.030 PMC5993597

[5] H. Cui , Y. Wang , H. Huang , et al., “GPR126 Protein Regulates Developmental and Pathological Angiogenesis Through Modulation of VEGFR2 Receptor Signaling,” Journal of Biological Chemistry 289, no. 50 (2014): 34871–34885. 25217645 10.1074/jbc.M114.571000 PMC4263886

[6] J. R. Heimall , D. Hagin , J. Hajjar , et al., “Use of Genetic Testing for Primary Immunodeficiency Patients,” Journal of Clinical Immunology 38, no. 5 (2018): 520–530. 10.1007/s10875-018-0489-8 29675737

[7] E. Dancy , P. Stratton , D. C. Pichard , et al., “Human Papillomavirus Disease in *GATA2* Deficiency: A Genetic Predisposition to HPV‐Associated Female Anogenital Malignancy,” Frontiers in Immunology 15 (2024): 1445711. 39267745 10.3389/fimmu.2024.1445711 PMC11390362

[8] S. Yu , M. S. Park , G. Y. Kim , et al., “Rare Case of Germline GATA2‐Deficiency With Merkel Cell Carcinoma and Acute Myeloid Leukemia,” Cancer Reports 7, no. 12 (2024): e70068. 39614632 10.1002/cnr2.70068 PMC11607133

[9] S. E. Turvey , F. A. Bonilla , and A. K. Junker , “Primary Immunodeficiency Diseases: A Practical Guide for Clinicians,” Journal of Allergy and Clinical Immunology 134, no. 3 (2014): 613–622. 20075404 10.1136/pgmj.2009.080630

[10] A. Bousfiha , L. Jeddane , C. Picard , et al., “The 2015 Update of the IUIS Phenotypic Classification for Primary Immunodeficiencies,” Frontiers in Immunology 5 (2014): 627. 29226301 10.1007/s10875-017-0465-8 PMC5742599

[11] D. T. Doan , P. V. Strebeck , A. D. Tran , et al., “Evaluation of Recurrent and Recalcitrant Warts in a Deaf Adolescent Male Reveals GATA2 Deficiency,” Journal of Allergy Clinical Immunology Global 3, no. 4 (2024): 100313. 39221430 10.1016/j.jacig.2024.100313 PMC11364118

[12] E. Karabiber and S. Baris , “Delineating the Clinical and Immunologic Characteristics: A Comparative Study of Inborn Errors of Immunity in Adult Versus Pediatric Diagnosed,” International Archives of Allergy and Immunology 185, no. 11 (2024): 1123–1135. 39226882 10.1159/000540538

[13] F. A. Bonilla , D. A. Khan , Z. K. Ballas , et al., “Practice Parameter for the Diagnosis and Management of Primary Immunodeficiency,” Journal of Allergy and Clinical Immunology 136, no. 5 (2015): 1186–1205.e2078. 26371839 10.1016/j.jaci.2015.04.049

[14] C. Moulin , B. Beaupain , F. Suarez , et al., “CXCR4 WHIM Syndrome Is a Cancer Predisposition Condition for Virus‐Induced Malignancies,” British Journal of Haematology 204, no. 4 (2024): 1383–1392. 38442908 10.1111/bjh.19373

[15] O. Shamriz , N. Zahalka , A. J. Simon , et al., “GATA2 Deficiency in Adult Life Is Characterized by Phenotypic Diversity and Delayed Diagnosis,” Frontiers in Immunology 13 (2022): 886117 Published 2022 May 6. 35603181 10.3389/fimmu.2022.886117 PMC9120659

[16] C. C. Homan , P. Venugopal , P. Arts , et al., “GATA2 Deficiency Syndrome: A Decade of Discovery,” Human Mutation 42, no. 11 (2021): 1399–1421. 34387894 10.1002/humu.24271 PMC9291163

[17] R. Bortnick , M. Wlodarski , V. de Haas , et al., “Hematopoietic Stem Cell Transplantation in Children and Adolescents With GATA2‐Related Myelodysplastic Syndrome,” Bone Marrow Transplantation 56, no. 11 (2021): 2732–2741. 34244664 10.1038/s41409-021-01374-y PMC8563415

